# Investigating impacts of and susceptibility to rail noise playback across freshwater fishes reveals counterintuitive response profiles

**DOI:** 10.1093/conphys/coaa089

**Published:** 2020-09-28

**Authors:** Ryan J Friebertshauser, Daniel E Holt, Carol E Johnston, Matthew G Smith, Mary T Mendonça

**Affiliations:** 1 Department of Fisheries, Aquaculture, and Aquatic Sciences, Auburn University, Auburn, AL 36849, USA; 2 Department of Biological Sciences, Columbus State University, Columbus, GA 31907, USA; 3 Department of Biological Sciences, Auburn University, Auburn, AL, 36849, USA

**Keywords:** Anthropogenic noise, bioacoustics, conservation, rail noise, stress

## Abstract

While the expansion of anthropogenic noise studies in aquatic habitats has produced conservation-based results for a range of taxa, relatively little attention has been paid to the potential impacts on stream fishes. Recent work has shown responses to road noise in single species of stream fish; however, assemblage-wide effects of anthropogenic noise pollution have not yet been investigated. By examining five metrics of disturbance across four ecologically and evolutionarily disparate species of stream fishes, a series of laboratory experiments aimed to describe the effects of and species susceptibility to anthropogenic noise playback. Each species studied represented a unique combination of hearing sensitivity and water column position. Physiological and behavioral metrics were compared across the presence and absence of rail-noise noise playback in four target species. Through repeated subsampling, the temporal dynamics of cortisol secretion in response to noise in two target species were additionally described. Rail-noise playback had no statistically significant effect on blood glucose or water-borne cortisol levels, with the exception of decreased cortisol in noise-exposed largescale stoneroller (*Campostoma oligolepis*). Time-course cortisol experiments revealed rapid secretion and showed minimal effects of noise at most observation points. The presence of noise produced significant changes in ventilation rate and swimming parameters in a portion of the four species observed representing the most conserved responses. Overall, effects of noise were observed in species contrary to what would be hypothesized based on theoretical hearing sensitivity and water column position demonstrating that predicting susceptibility to this type of stressor cannot be accomplished based off these course considerations alone. More importantly, we show that anthropogenic noise can disrupt a variety of behavioral and physiological processes in certain taxa and should be further investigated via measures of fitness in the wild.

## Teaser

This study investigated the effects of railway noise across distinct freshwater fishes to predict species-specific susceptibility as well further define noise as an environmental detriment. Results revealed that while predicting susceptibility is difficult, the impacts experienced further distinguish anthropogenic noise as a relevant stressor to the species effected.

## Introduction

As global transportation increases, it is estimated that by 2050, road and railway lengths will expand by 60% (Dulac, 2013). This projection is especially concerning given that US road miles are currently almost equal in length to that of stream and river miles (Riitters and Wickham, 2003). The expansion of transportation infrastructure is a known contributor of light pollution, chemical loading, the formation of movement barriers and habitat modification (Van der Ree *et al.*, 2015), but a relatively understudied aspect is its role in the production of anthropogenic noise. Anthropogenic noise can be defined as any human-generated sounds that may be detrimental to an organism (Slabbekoorn *et al.*, 2010). Beyond the civilian road, other potential sources of such noise include artificial waterways, trails, railroads and utility easements. Research on terrestrial organisms exposed to noise has shown a reduction in available habitat (Schaub *et al.*, 2008; Blickley *et al.*, 2012a; Ware *et al.*, 2015), alterations of communication signals (Vargas-Salinas *et al.*, 2014; Templeton *et al.*, 2016) and indicators of physiological stress (Blickley *et al.*, 2012b; Tennessen *et al.*, 2014; Green *et al.*, 2015). However, the consequences brought on by noise in these systems are not limited to terrestrial habitats.

While a large proportion of work concerning anthropogenic noise and aquatic organisms has focused on marine mammals (Cox *et al.*, 2016), attention towards fishes has steadily grown over the years (Popper and Hawkins, 2019). This expansion is especially important given that acoustic perception is of great importance to spawning (Holt and Johnston, 2014a), predator detection (Mann *et al.*, 1997), foraging (Holt and Johnston, 2011), competitive interactions (Amorim and Hawkins, 2000) and more in a number of fishes (Handegard *et al.*, 2003; Vasconcelos *et al.*, 2007; Purser and Radford, 2011; Sebastianutto *et al.*, 2011; Bruintjes and Radford, 2013; Simpson *et al.*, 2016). In addition to impacting critical behaviors in fishes, noise exposure has proven to affect certain physiological processes, most commonly observed through the increase of circulating glucocorticoids and energy substrates that are common indicators of a primary stress response (Wysocki *et al.*, 2006; Buscaino *et al.*, 2010; Celi *et al.*, 2016).

Freshwater fishes show commonalities with marine species in regard to the importance of acoustic perception and the impact noise has upon it, although the sources of noise often differ. While anthropogenic noise in marine habitats is produced by shipping vessels (Wysocki *et al.*, 2006), navy-operated sonar systems (Halvorsen *et al.*, 2012) and pile driving (Mueller-Blenkle *et al.*, 2010), noise in streams is primarily generated by roads (Riitters and Wickham, 2003; Ware *et al.*, 2015; Castaneda *et al.*, 2020). Additionally, different bridge structures vary in the types of noise they emit. While heavily trafficked automobile bridges produce a more constant noise, signals from railway bridges can be characterized by higher amplitude, shorter durations (Halfwerk *et al.*, 2011) and more sporadic occurrences. With this, the two sources can be thought of as chronic and acute stressors, respectively. To further complicate management of this disturbance, studies comparing susceptibility to noise across species are limited.

In regards to hearing in fishes, species are coarsely divided across a conceptual continuum based around sensitivity to sound pressure with more sensitive taxa possessing anatomical adaptations that bring the inner ear into close proximity to a pressure-to-mechanical displacement converter such as an air bubble (Popper and Fay, 2011). One example of these adaptations is the presence of Weberian ossicles, a synapomorphy of the superorder Ostariophysi, which connects the swim bladder (air bubble) to the inner ear via modified vertebrae. Based on Popper and Fay’s work (2011), fishes with this specific adaptation possess the greatest hearing sensitivity on the proposed hearing continuum. Species lacking an air bubble are theoretically incapable of detecting the pressure component of sound, thereby placing them at the least sensitive end of the continuum. Species lacking specialized anatomy (largemouth bass (*Micropterus salmoides*)) (Graham and Cooke, 2008), and even an air bubble (blackfin darter (*Etheostoma nigripinne*) and fringed darter (*Etheostoma crossopterum*)) (Johnston and Johnson, 2000), however, are still able to detect the particle motion component of sound through direct stimulation of the ear, the lateral line and superficial neuromasts. These results alone suggest that species-specific susceptibility to anthropogenic noise requires further investigation. A further complication to predicting susceptibility is the ability of certain benthic fishes to detect substrate vibrations (Janssen, 1990). This suggests that individuals in contact with the benthos may be simultaneously impacted by both the pressure and vibratory components of anthropogenic noise, theoretically culminating in a more severe stressor.

Holt and Johnston, (2014b) were one of the first to document the impacts of anthropogenic noise on small, stream fishes. This work illustrated that in the presence of road noise, blacktail shiner (*Cyprinella venusta*) increased the amplitude of vocalizations in order to effectively transmit signals to conspecifics (i.e. the Lombard effect) (Holt and Johnston, 2014b). Further research on blacktail shiner revealed that the presence of road noise leads to a significant increase in hearing thresholds and levels of the primary stress hormone cortisol (Crovo *et al.*, 2015). While these studies document clear, detrimental impacts to this species, the diversity of fishes studied must expand in order to better represent native fish communities. In line with a 2016 meta-analysis (Cox *et al.*, 2016), we additionally see the need for measurement of both behavioral and physiological disturbances when attempting to quantify the effect of noise. While this integrative approach has been used to study the impacts of anthropogenic noise on fish (Buscaino *et al.*, 2010), work concerning multiple, sympatric stream species is rare (Radford *et al.*, 2016).

This study examined the physiological and behavioral effects of acoustic pollution propagated by an active railway bridge on four ecologically and evolutionarily distinct species of sympatric stream fishes that differ in auditory anatomy and water column position. Data were gathered to better understand the impacts of noise on species that represent a common fish community and if proposed hearing sensitivity and/or microhabitat use can predict species-specific susceptibility. Through two manipulative laboratory experiments, five indices of disturbance were measured in blacktail shiner, bluegill sunfish (*Lepomis macrochirus*), largescale stoneroller and redline darter (*Etheostoma rufilineatum*). Blood glucose, water-borne cortisol, ventilation rate, average swimming velocity and the elicitation of a startle response in the presence and absence of rail noise playback were measured across all species. Additional trials quantifying noise-induced cortisol secretion at multiple time points in blacktail shiner and bluegill sunfish were conducted in order to better elucidate the temporal dynamics of the glucocorticoid stress response in these fishes. Based on proposed hearing sensitivities and location in the water column, we hypothesized that detrimental shifts in the indices measured would be most prevalent in the benthic Ostariophysan largescale stoneroller due to their location at the upper end of the hearing continuum and contact with substrate vibrations. Inversely, we hypothesized that the pelagic, non-Ostariophysan bluegill sunfish would be least affected. Our ultimate goals were to investigate impact of noise on these taxa and relate susceptibility to the course distinctions described above in order to simplify future management efforts.

**Table 1:** Matrix describing combinations of proposed hearing sensitivity and water column position unique to each study species. Ostariophysans represent the upper end of Popper and Fay’s (2011) proposed continuum of hearing sensitivity

	Ostariophysan	Non-Ostariophysan
Pelagic	Blacktail shiner	Bluegill sunfish
Benthic	Largescale stoneroller	Redline darter

## Methods

### Fish collection and husbandry

Each target species fulfilled a unique combination of proposed hearing sensitivity (Ostariophysan or non-Ostariophysan) and water column position (pelagic or benthic) (Table 1). Due to our goal of predicting impact based upon course and obvious distinctions across these taxa, neither hearing sensitivity nor water column position was quantified. Individuals were collected via seine between May and June 2018 from Chewacla Creek (Lee County, AL), Auburn University Fisheries Pond 15 (Lee County, AL), Middle Cypress Creek (Lauderdale County, AL) and Shoal Creek (Lauderdale County, AL). All animals were collected at a distance of at least 100 stream meters away from any bridge crossing to control for previous exposure.

Fishes were transported to the lab in coolers containing aerated stream water and housed in multiple, 68-l aquaria with identical vegetation, diet (*Chironomid* spp.) temperature (22°C), diel period (12,12) and ceramic cover tiles. All housing tanks were filtered using an exterior, hanging carbon filter. Fishes were acclimated to housing tanks for at least 24 h prior to testing. This project was covered by Auburn University animal protocol number 2017-2333.

### Anthropogenic noise acquisition

The anthropogenic noise playback used throughout this research was recorded at a beam-style bridge crossing Red Creek (Macon County, Alabama) during the passing of a train. A hydrophone (Hi-tech HTI-96-MIN, sensitivity −164.4 re 1 V/μPa, frequency response: 0.002–30 kHz) connected to a digital recorder (Marantz PMD 661) was placed 7.3 m upstream of the center bridge pile and 8 cm above the stream bed (water depth = 18 cm) to record the pressure component of the rail noise. A geophone (PCB Piezotronics Model 608A11/030 AC, sensitivity = 10.4 mV/g), which was routed through a signal conditioner set to 1× gain (PCB Piezotronics Model 480B21) and connected to a digital recorder (Tascam DR70MKII) was placed adjacent to the microphone and recorded the substrate vibration component of the train passing. The geophone was glued to a rock and set into the substrate to most efficiently transfer energy from the benthos to the sensor.

**Figure 1:**
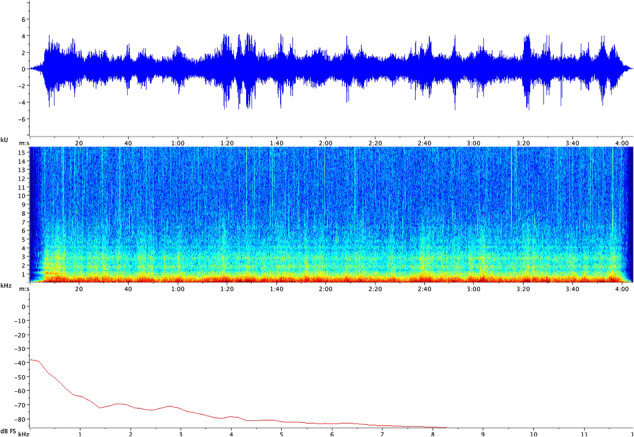
Waveform (top), spectrogram (middle) and power spectral density (bottom) visualization of the rail-noise playback used throughout this study.

Field recordings were edited to only include the duration of the train crossing the bridge (4 min 2 s) and bound by a 3 s fade in and fade out (Fig. 1). Both pressure and vibratory components of playbacks were calibrated to reflect natural levels by recording in-tank playbacks, using the same equipment and settings used in the field and adjusting the amplitude as needed until the amplitude of the waveform approximated that of the audio recorded in the field. While the reverberation and resonance caused by any artificial testing environment lead to imperfectly simulated playback (Akamatsu *et al.*, 2002), the observed differences in sound pressure between control and noise conditions verified our stimulus as adequately novel. In-tank levels were compared with field recordings in version 1.4 of Raven Pro software (Cornell University, Ithaca, NY). All sound editing was completed using version 2.2.1 of Audacity® recording and editing software (Mazzoni, 1999).

### Physiological experiment

Blood glucose concentration, waterborne cortisol levels and ventilation rate were independently collected from 30 individuals from each species in the presence (*n* = 15) and absence (*n* = 15) of the noise playback. Fishes were tested individually in a 700-ml glass collection dish filled with 500 ml of dechlorinated tap water. Collection dishes were partly submerged in a 200-l aquarium and positioned 6.5 cm from a horizontally oriented UW30 speaker (Universal Sound Inc., Oklahoma City, OK) connected to a SLA1 studio amplifier (Applied Research and Technologies). Unlike behavioral experimentation, this testing environment did not allow for the replication of substrate vibrations. All physiological experiments were conducted between 1700 and 2200 h to control for diel variations in cortisol and glucose.

Fishes were netted from housing tanks, placed in a 1-l transfer beaker and relocated to the testing environment. Individuals were then exposed to either a 30-min control period (silence) or a 30-min playback stimulus consisting of three, full train crossings equally interspersed with silence. Collection dishes were topped with a thin mesh throughout the trial to prevent fish from escaping into the surrounding water. Ventilation rate was recorded using a GoPro HERO4 (GoPro, Inc., San Mateo, CA) positioned outside of the tank. Opercular beats were tallied in 10 s increments immediately after the onset of the last two train crossing playbacks or where they would have occurred in the case of control trials. Tallies were transformed to beats per minute and individual results were averaged. Ventilation rate during the first noise playback was omitted from analysis to eliminate the likely increase in ventilation caused by transfer from housing to test tank.

Immediately following the trial, individuals were anesthetized with 75 mg/l of Tricaine methanesulfonate (MS 222) in order to measure blood glucose concentrations. After stage three anesthesia was reached, defined as loss of equilibrium and cessation of locomotion (Schoettger and Julin, 1969), ~0.05 ml of blood was drawn using a heparinized, 32-gauge insulin syringe (MHC Medical Products, LLC, Fairfield, OH) and placed on the test strip of an Accu-chek Compact Plus glucometer (Roche Diagnostics Corp., Indianapolis, IN). Where relative measurements of blood glucose concentrations are of interest, portable glucometers have proven to be an accurate and efficient alternative to other methods when studying fishes (Beecham *et al.*, 2006). Blood glucose was measured <5 min post-trial.

Water-borne cortisol was sampled by filtering 500 ml of holding water through a grade 2 Whatman paper filter (GE Healthcare UK Limited, Buckinghamshire, ENG) then a primed C-18 cartridge (Sep-pak, Waters Technology Corporation, Milford, MA). Cartridges were primed with two washes of 100% EtOH followed by two washes of deionized water. All holding dishes and filtering equipment were sterilized with 100% EtOH between trials. Cartridges were stored at −20°C prior to analysis. Free cortisol was eluted from the cartridges with two 2-ml washes of 99.5% ethyl acetate followed by evaporation under a stream of nitrogen gas. Dried residues were resuspended in 500 μl of enzyme immunoassay (EIA) buffer and further diluted with EIA buffer to allow for accurate analysis with the ELISA kit used (Cayman Chemical, Ann Arbor, MI). This particular assay and protocol were previously validated by Crovo *et al.* (2015).

### Temporal dynamics of cortisol secretion

Our inconclusive findings concerning cortisol secretion in response to noise in the physiological studies described above prompted us to investigate the temporal dynamics of cortisol secretion at a finer resolution. For this portion of the study, we chose to include the blacktail shiner due to previously observed cortisol secretion in the presence of noise (Crovo *et al.*, 2015) and bluegill sunfish as a comparative ‘non-Ostariophysan’. Nine individuals of each species were exposed to both noise and control treatments. The noise playback, testing environment, tank transfer procedure and trial duration used in this experiment were identical to that of the initial physiological experiment. To quantify cortisol changes over time, holding water was subsampled at three fixed, time steps (5, 15 and 30 mins) over the 30-min trial using a novel collection procedure. No samples were collected at 0 min due to the assumed amount of hormone being zero.

Subsampling was accomplished by fitting five vinyl tubes inside the collection dish that were each attached to a 60 cc, catheter-tipped syringe. At each time step, a 40-ml subsample of holding water was withdrawn into the syringe and filtered outside of the testing room using the same protocol of the initial physiological experiment. Reduction of water levels in housing aquaria has been shown to elicit a neuroendocrine stress response (Einarsdóttir and Nilssen, 1996); therefore, we preloaded two syringes with 51 ml of dechlorinated tap water (40 ml of subsample + 11-ml volume of tube) to immediately replace the water removed at each sample point. Syringes were used for a single sample and all other collection equipment was sterilized with 100% EtOH between trials.

The elution and assay protocol for all samples was identical to that of the initial physiological experiment. To correct for the dilution of holding water caused by water replacement at the second (8.7% dilution) and third time step (16.6% dilution), cortisol concentrations at these time steps were multiplied by 1.087 and 1.166, respectively.

### Behavioral experiment

Two metrics of swimming parameters were measured in 15 individuals from each species in the presence and absence of noise playback. Swimming behavior was recorded from a GoPro HERO4 suspended 2.1 m above a 1022-l mesocosm at 60 frames per second. The experimental mesocosm included 15 cm of rinsed sand as substrate with a water depth at a relatively shallow 23 cm above the substrate to minimize potential distortion of results caused by vertical swimming. The noise stimulus was presented through a SLA1 studio amplifier leading to two UW-30 underwater speakers. One speaker was mounted horizontally in the water column on the far-right hand side of the mesocosm 1 cm off the substrate and the other was buried face up, 3 cm below the substrate, oriented toward the surface, directly in front of the horizontally oriented speaker to reproduce substrate vibrations. Speakers were independently amplified to better replicate the natural pressure and vibratory components of the playback. A disconnected speaker was vertically mounted to the far-left side of the mesocosm to avoid potential side bias.

Fishes were observed individually and given a 15-min acclimation period in the testing mesocosm. After acclimation, video recording was remotely engaged using the GoPro mobile app. Behavioral trials consisted of a 4 min and 2 s period of silence immediately followed by the rail-noise playback of same length. Video recording was remotely terminated at the end of the noise playback, resulting in an 8 min and 4 s testing period. All behavioral trials were conducted between 1000 and 1400 h. Tank transfer protocols were identical to the physiological experiment.

Prior to movement analysis, behavioral videos were processed and converted into 1-fps image sequences using a custom command line script (www.github.com/rfriebertshauser/auburn-fish-biodiversity). Fish position was tracked at each frame using the AnimalTracker plugin (Gulyás *et al.*, 2016) of the open-source image analysis program Fiji (Schindelin *et al.*, 2012). The sequential XY coordinates produced from this tracking procedure were then used to calculate average swimming velocity (cm/s/trial) for control and noise exposure periods. The elicitation of a startle response was quantified by comparing swimming velocity across three, 10-s time periods surrounding the onset of noise playback. Time periods included 10 s immediately before the onset of noise, 10 s during the initial fade in sequence of the playback and 10 s of full-signal noise and were termed ‘pre-noise’, ‘fade-in’ and ‘full-signal’, respectively.

### Statistical analyses

The effect of treatment on blood glucose, cortisol concentrations and ventilation rate was tested using general linear models that included species, treatment, total length and the interactive effects of treatment by species. To analyze interaction contrasts not presented in the standard table of coefficients for the physiological models above, a general linear hypothesis test was used. Separate models using only total length and treatment were then used to compare the effect of noise within each species. Water-borne cortisol concentrations were log10 transformed to achieve normality of residuals.

**Figure 2:**
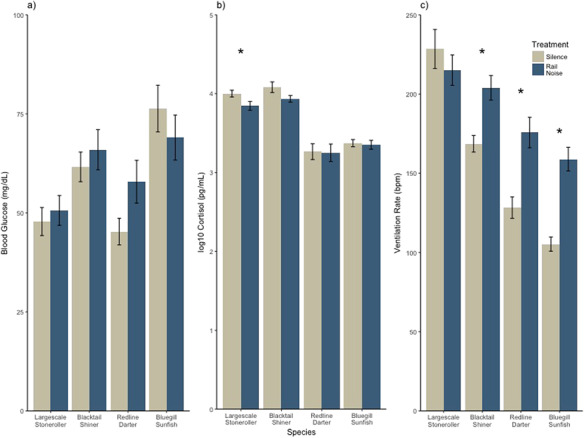
Physiological metrics of disturbance across rail-noise and silence-exposed individuals from each species. Presented as mean ± SE. Asterisks indicate statistical significance.

To better understand the temporal dynamics of cortisol secretion in blacktail shiner and bluegill sunfish, the effect of treatment and time on water-borne cortisol concentrations were tested using a general linear mixed model that included species, total length, time step, treatment, a three-way interaction between treatment, time step and species, and ID. ID acted as a random effect to eliminate pseudoreplication caused by the repeated measure design of this experiment. Additional models including treatment, time step, total length, a treatment by time-step interaction term and a random effect of ID were used to investigate the interactive effect of treatment and time within each species.

The effect of treatment on average swimming velocity was tested with a general linear mixed model that each included species, treatment, total length, interactive effects of treatment by species and ID as a random effect to control for pseudoreplication. To test for the elicitation of a startle response, the same model was used with the exception of treatment being replaced with the 10-s time periods. To analyze interaction contrasts not presented in the standard table of coefficients a general linear hypothesis test was used. Models using only total length and treatment (or 10-s time period) were then used to compare the effect of noise within each species.

All statistical analyses were conducted using R version 3.3.4 (R Core Development Team, 2016) with an alpha criterion of 0.05. Data are reported as effect size +/− standard error (SE). All models were tested for goodness of fit against a null model using either a likelihood ratio test for general linear models or by comparing AIC scored produced by mixed effects models. Normality of data was observed visually from residual-fitted plots. All figures were produced using the R package ggplot2 (version 3.2.1) (Wickham, 2016).

## Results

### Physiological metrics

Blood glucose levels did not significantly shift in the presence of rail-noise playback in blacktail shiner, largescale stoneroller, redline darter or bluegill sunfish (*P* = 0.51, 0.43, 0.11 and 0.42) (Fig. 2) Additionally, no significant interactions were present in the full model indicating no difference in cortisol change, with respect to treatment, among any of the four species.

Waterborne-cortisol concentrations were not significantly affected by the presence of noise playback in blacktail shiner, redline darter or bluegill sunfish (*P* = 0.079, 0.99 and 0.80) and no significant interactions were present. Largescale stoneroller, however, exhibited a 0.15 (+/− 0.071 SE) log10 pg/ml decrease in response to noise treatment (*P* = 0.041) (Fig. 2).

The model used to test the effects of rail-noise treatment on ventilation rate across species produced significant interactions between species and treatment such that ventilation rate of blacktail shiner, redline darter and largescale stoneroller shifted by 49.11 (+/−16.62 SE), 63.73 (+/−16.76 SE) and 68.40 (+/−16.65 SE) bpm more in the presence of rail-noise compared to the same treatment in largescale stoneroller (*P* = 0.0038, 0.00024 and 7.77e-05). Within-species analysis revealed that in the presence of noise playback blacktail shiner, redline darter and bluegill sunfish increased ventilation rate by 35.30 (+/−9.49 SE), 53.13 (+/−12.033 SE) and 58.22 (+/−8.63 SE) bpm compared to control treatment (*P* = 0.00092, 0.00015 and 7.44e-07). Largescale stoneroller showed no statistically significant difference in ventilation rate between treatment and control conditions (Fig. 2).

**Figure 3:**
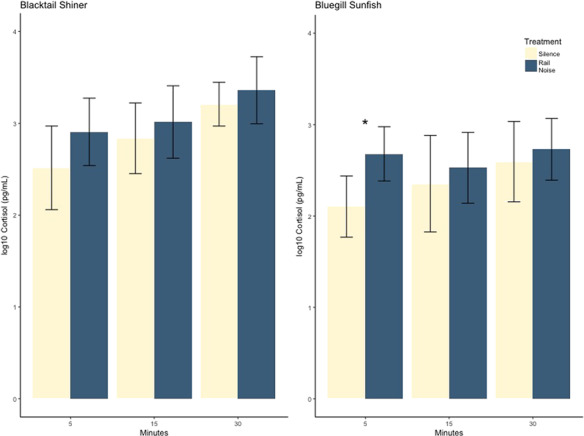
Log10 water-borne cortisol concentrations collected at 5, 15 and 30 min from blacktail shiner and bluegill sunfish. Presented as mean ± SE. Asterisk denotes statistical significance.

Time-course trials investigating temporal dynamics of cortisol in blacktail shiner and bluegill sunfish revealed no significant interactions between species, time step and treatment; indicating no difference in cortisol change with respect to the three variables. Species-specific models additionally produced no significant interactions between time step and treatment but revealed that bluegill sunfish exposed to noise secreted 0.57 (+/−0.19 SE) log10 pg/ml more cortisol than the control group at the 5-min time step (*P* = 0.024). No significant differences were found across any species, time step or treatment. (Fig. 3).

### Behavioral metrics

**Figure 4:**
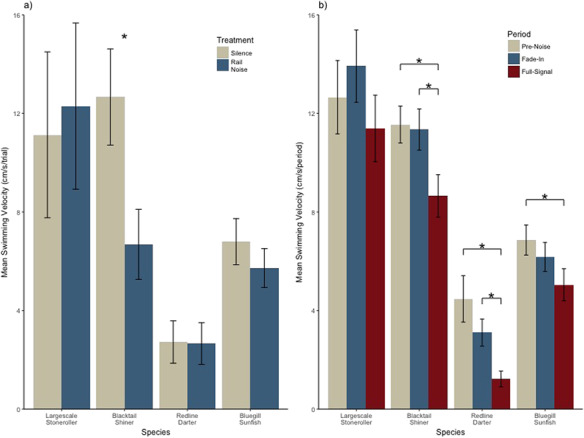
Behavioral metrics of disturbance across rail-noise and silence-exposed individuals from each species. Presented as mean ± SE. Asterisks denote statistical significance.

The model used to test the effects of rail noise on average swimming velocity produced significant interactions such that the average swimming velocity of largescale stoneroller, redline darter and bluegill sunfish shifted 7.15 (+/−1.76 SE), 5.92 (+/−1.76 SE) and 4.91 (+/−1.76 SE) cm/s less in the presence of noise compared to the same treatment in blacktail shiner (*P* = 0.00020, 0.00079 and 0.0054). Within-species analysis revealed that in the presence of noise playback, blacktail shiner decreased average swimming velocity by 5.98 (+/−1.87 SE) cm/s compared to control treatment (*P* = 0.0064) (Fig. 4).

The full model used to test the elicitation of a startle response due to rail-noise treatment produced no statistically significant interactions between species and treatment. Within-species analysis revealed that during the full-amplitude time period, blacktail shiner, redline darter and bluegill sunfish significantly decreased swimming velocity by 2.9 (+/−0.87 SE), 3.25 (+/−0.82) and 1.81 (+/−0.74 SE) cm/s compared to the pre-noise time-period (*P* = 0.0010, 0.00010 and 0.015). Blacktail shiner and redline darter additionally exhibited significantly decreased swimming velocity by 2.69 (+/−0.87 SE) and 1.88 (+/−0.82 SE) cm/s during the full-amplitude time period compared to the fade-in time period (*P* = 0.0021 and 0.023) (Fig. 4). No significant differences in swimming velocity between the pre-noise and fade-in time periods were observed (Table 2).

## Discussion

This study examined the physiological and behavioral responses of four species of stream fishes exposed to anthropogenic noise playback to gain insight on species-specific disturbance profiles and how they may serve as indicators of individual and assemblage-wide fitness consequences. Additional experimentation aimed to better describe the temporal profile of cortisol secretion in two target species exposed to rail noise. Our results showed both behavioral and physiological responses to noise playback; however, our hypotheses surrounding species-specific susceptibility to noise based on traditional classification of hearing sensitivity and water column position were unsupported.

In the presence of noise playback, we observed no significant change in blood glucose concentrations in any of the four species. While these results are inconsistent with those seen in the literature (Wysocki *et al.*, 2007; Celi *et al.*, 2016; Vazzana *et al.*, 2017), it should be noted that the mobilization of glucose serves as a secondary stress response and is often exhibited over longer periods of time than our study observed. Additionally, the caudal vasculature in these small fishes presented difficulty not only in acquiring the requisite volume but also in making sure surrounding bodily fluids did not dilute the blood sample. We believe that increasing the duration of observation and a refinement of blood sampling technique would better allow for the observation of this response.

The presence of rail noise in our initial experimentation induced no significant changes in water-borne cortisol concentrations with exception of a decrease in noise-exposed largescale stoneroller, potentially caused by the silence of control trials being more stressful compared to the acoustic environment of home streams. This lack of effect is contrary to examples present in the literature (Ellis *et al.*, 2004; Wysocki *et al.*, 2006; Fanouraki *et al.*, 2008; Kammerer *et al.*, 2010) and most importantly are contrary to a 2015 study that, through the use of a similar protocol followed in this work, observed a significant effect of noise on blacktail shiner cortisol concentrations (Crovo *et al.*, 2015). However, our time-course trials investigating the temporal dynamics of stress-induced cortisol secretion in blacktail shiner and bluegill sunfish suggest further complexity to this primary stress response and offer explanation as to why our results diverged from that of previous studies.

Observations from time-course experimentation show that the lack of effect observed in our initial experiment was a combined artifact of temporal sampling fidelity and testing methodology. The only significant effect of noise was observed in bluegill sunfish at the 5 min time step. While we did not observe any other effects of time on cortisol, this result describes a rapid secretion of cortisol previously seen in the literature (Foo and Lam, 1993; Pottinger and Moran, 1993; Ramsay *et al.*, 2009; Koakoski *et al.*, 2012) and highlights the need for sampling across time when investigating neuroendocrine responses to stress. We posit that no significant differences were observable in the initial experiment as well as the 30-min time step of the time-course study due to netting and tank transference acting as an equivalent stressor in control groups. Stress induced by testing procedures have been noted in the literature (Pickering *et al.*, 1982; Wong *et al.*, 2008) and raise concerns around how collection of cortisol is conducted due to fishes sensitivities to a wide array of disturbances (Foo and Lam, 1993). While the response observed in bluegill sunfish is contrary to our hypothesis, a variety of species occurring at the lower end of Popper and Fays’s (2011) proposed hearing continuum have been observed mounting a neuroendocrine response to noise (Wysocki *et al.*, 2006; Mueller-Blenkle *et al.*, 2010; Nichols *et al.*, 2015).

We posit that the lack of treatment effect in blacktail shiner could be explained by the presence of Schreckstoff, a conspecific alarm cue known to induce cortisol secretion in the superorder Ostariophysi (Rehnberg *et al.*, 1987), in the holding tank from which individuals were sourced. Foo and Lam (1993) note that netting from a holding tank not only serves as an additional stressor to targeted individuals but also can impact cohabitating individuals as well. A communal stress response such as this is more likely if the species of interest secretes an alarm cue. We therefore hypothesize that netting and tank transference induced the secretion of Schreckstoff in blacktail shiner and raised cortisol concentrations of cohabitating individuals to near maximal levels prior to observation thereby masking the effect of noise exposure during trials. Depending on the tank transference methods used by Crovo *et al.* (2015), this theory may explain the discrepancies between this study and ours. Acclimation to testing environments may serve to mitigate this artifact; however, the necessity of sampling water-borne cortisol in relatively small testing environments will make this difficult.

The observed increase in ventilation rate, in all species with the exception of largescale stoneroller, reflects findings in the literature concerning increased ventilation by noise (Nedelec *et al.*, 2016) and other stimuli (Gibson and Mathis, 2006; Zhao *et al.*, 2017). Along with the elicitation of a startle response, increased ventilation was present in more species than any other metric suggesting that this response may be most conserved and rapidly induced across a wide range of species. While ventilation rate serves as a reliable proxy for metabolic alteration, further studies should seek to comparatively assess specific metabolic impacts brought on by increased ventilation in the presence of noise.

Alterations of swimming parameters are commonly induced by noise (Ona and Godø, 1990; Sarà *et al.*, 2007; Holles *et al.*, 2013); however, the direction of change can vary and appears to be species specific (Kastelein *et al.*, 2007). In this study, only blacktail shiner decreased average swimming velocity in the presence of noise. However, we did observe the elicitation of a startle response in all species with the exception of largescale stoneroller. The observed short-term reduction of swimming activity represents a fairly conserved, acute response to noise followed by habituation in redline darter and bluegill sunfish, supported by a lack of change in swimming activity over the entirety of their trials. While redline darter lacks a gas bladder, which suggests a lack of sensitivity to the pressure component of sound, we posit that these individuals may have reacted to substrate vibrations presented in the testing mesocosm, which has been seen in other benthic species (Abboud and Coombs, 2000).

While largescale stoneroller is a member of the clade Ostariophysi and has been observed responding to acoustic stimuli (Holt and Johnston, 2011), this study presents limited evidence of disturbance, with the exception of decreased cortisol concentrations in the presence of rail-noise playback. Similarly, unexpected results have been observed in other comparative, acoustics studies (Kastelein *et al.*, 2008) and suggest that quantification of hearing sensitivity as well as responses to standard stressors be conducted to explain these results.

**Table 2:** Results summary for physiological and behavioral experiments. Bolded results indicate statistically significant effect of noise.

		Blacktail shiner	Largescale stoneroller	Redline darter	Bluegill sunfish
Blood glucose (mg/dl)	Effect size *P*-value SE	4.34 0.51 6.42	3.77 0.43 4.69	11.16 0.11 6.76	−6.85 0.42 8.38
Water-borne cortisol (log10 pg/ml/30 min)	Effect size *P*-value SE	−0.15 0.079 0.08	**−0.15** **0.041** **0.071**	−0.0016 0.99 0.16	−0.064 0.371 0.069
Ventilation rate (bpm)	Effect size *P*-value SE	**35.30** **0.000092** **9.49**	−13.07 0.439 16.61	**53.13** **0.00015** **12.03**	**55.22** **7.44E-07** **8.63**
Average swimming velocity (cm/s/trial)	Effect size *P*-value SE	**−5.98** **0.0064** **1.87**	1.16 0.43 1.42	−0.062 0.85 0.33	−1.072 0.18 0.77
Startle response (pre-noise to full-amplitude) (cm/s/10s)	Effect size *P*-value SE	**−2.89** **0.001** **0.87**	−1.27 0.37 1.43	**−3.25** **0.00010** **0.82**	**−1.81** **0.0150** **0.74**
Startle response (fade-in to full-amplitude) (cm/s/10s)	Effect size *P*-value SE	**−2.69** **0.0021** **0.87**	−2.53 0.077 1.43	**−1.88** **0.023** **0.82**	−1.13 0.13 0.74

Bolded values indicate a statistically significant effect of noise on each species.

### Indicators of potential fitness consequences

Behavioral and physiological performance can be linked to fitness through a network of relationships. While direct measurements of fitness were not taken in this study, the disturbance responses observed may act as indicators of potential fitness consequences thereby inspiring avenues for future investigation.

The rapid neuroendocrine stress response seen in bluegill sunfish can be viewed as a response to an emergency situation, resulting in brief energy limitation (Schreck, 2010) and alteration of behavioral performance (Algera *et al.*, 2017). Although this effect was transitory, it is possible that rail noise concurrent with additional environmental stressors may serve to allocate energy or attention away from short-term tasks. The observed increase in ventilation rate of blacktail shiner, redline darter and bluegill sunfish is suggestive of an increase in metabolism (Millidine *et al.*, 2008), which leads to the reallocation of energy away from growth (Barton and Iwama, 1991) to maintenance of the current stressor (Santos *et al.*, 2010). The impacts caused by this reallocation of energy may have even stronger implications to the fitness of developing or reproductive fishes. While the chronic reduction of swimming behavior in blacktail shiner and acute reduction in redline darter and bluegill sunfish suggest a temporary decrease in energy expenditure, alterations in motility present multiple fitness consequences at the behavioral level such as decreased foraging (Voellmy *et al.*, 2014) or reproductive success. Blacktail shiners are known to actively fight for access to nesting crevices (Heins, 1990). An overall reduction in motility could likely impair this critical behavior thereby compromising access to nesting sites. Unlike blacktail shiner, redline darter and bluegill sunfish both engage in parental care through nest guarding (Etnier and Starnes, 1993), which, in some species, requires diligent swimming to be upheld (Cooke *et al.*, 2002). Environmental stressors have also been seen to cause altered nesting behavior (Bruintjes and Radford, 2013) as well as decreased parental investment in young (Algera *et al.*, 2017) potentially impacting reproductive success. Furthermore, bluegill sunfishes’ behavior of nesting in densely packed nest colonies (Boschung and Mayden, 2004) suggests impact to reproduction at an unusually large scale. While measuring long-term impacts to reproduction and survival in the presence of noise may be difficult, the metrics of disturbance measured in this study highlight a range of potential ramifications at the fitness level.

### Conservation Implications 

The 2018 Living Planet Report, prepared by the World Wide Fund for Nature, cites that 45% of current abundance decline in freshwater fishes is attributable to habitat degradation in Nearctic fishes. This combined with the predicted global addition of 335 000 km of rail lines by 2050 (Dulac, 2013) demonstrates the relevance of this work. Unlike previously studied traffic noise, the temporal structure of rail noise may present a more complex stressor. One tactic fishes may use to mitigate these stressors, which has been observed in marine species (Handegard *et al.*, 2003; Mueller-Blenkle *et al.*, 2010), is to simply avoid disturbed habitat (Schreck *et al.*, 2001). However, stream fishes exist in a linear world and especially individuals restricted to specific microhabitats may not have this option. In addition to noise, this reduction in potential distribution inherently amplifies the impacts of other environmental stressors as well, which are thought to act cumulatively upon the animals affected (Schreck, 2000). Perhaps most importantly, it is thought that community disturbances such as trophic uncoupling could result from anthropogenic noise (Jacobsen *et al.*, 2014), thereby affecting systems at an assemblage-wide level.

## Conclusions

Our results suggest that the relationship between taxa, water column position and susceptibility to noise are not clear and, therefore, should be investigated from a more thorough, mechanistic perspective involving the production of audiograms (descriptions of hearing sensitivities) in species studied. While most metrics of disturbance in these experiments were not marked in their effects, the results of this study suggest that future work focus on the most conserved responses reported above (changes in ventilation rate and swimming parameters). These results combined with the known diversity of hearing in fishes (Popper *et al.*, 2003) echo the sentiments of a review on hearing capability in fishes (Popper and Fay, 2011) and that studying anthropogenic noise using the ‘generalist’/‘specialist’ framework will impede research seeking conservation goals in this field. Future work should seek to find correlates between these indicators and measurements of survival and reproductive success in freshwater fish communities at environmentally relevant spatial and temporal scales.

## Funding

This work was supported by the Fisheries, Aquaculture and Aquatic Sciences Department of Auburn University.
